# Institutionalised Commitment and Its Origins

**DOI:** 10.1007/s12110-026-09515-2

**Published:** 2026-03-28

**Authors:** Saira Khan

**Affiliations:** 1 Department of Philosophy, University of Bristol, Cotham House, Bristol, BS6 6JL UK; 2 Department of Philosophy, Lingnan University, Ho Sin Hang Building, Tuen Mun N.T., Hong Kong

**Keywords:** Commitment, Institution, Neolithic, Evolution, Cooperation

## Abstract

Schelling (1960) and Frank (1988) famously offered commitment as an explanation of the stability of human cooperation in the face of incentives to cheat. Reputationally-enforced commitments were part of the explanation for human cooperation for much of our history (Khan, 2024, 2025). In this article, I consider the origins and effects of *institutionalised* commitments. These are commitments which are contractually enforced by third parties, rather than only reputationally enforced. I discuss how these commitment offer advantages for securing cooperation over and above our previous forms of commitment. I then offer an account of their potential origins. I suggest that a change in our cooperative landscape and organisational structure occurring in the Neolithic -- in particular, the rise of agricultural economies and hierarchical society -- opened the door to a new means of enforcement for commitments, affecting the success of our cooperative practices.

## Introduction

Cooperative interaction between two or more individuals can lead to synergies which could not have been achieved by agents in isolation. For example, a group of lionesses can kill a deer when one alone may not be able to shepherd the target away from her herd. Social insects achieve synergies through building protective structures to benefit the whole group. Division of labour, seen frequently between the sexes in tribal communities, also allows for collective profits as time can be spent equally on different tasks and expertise in one skill is easier to build than expertise in many. Cooperation can also manage risk through the reciprocal sharing of resources within a group when one agent may be incapacitated by injury or illness. Humans cooperate in a number of different ways and cooperation is often spontaneous, even proactive, between individuals who have never interacted before.

Cooperative behaviours can be altruistic or mutually beneficial.^1^ Typically, we refer to altruistic behaviours as those which benefit the recipient of the behaviour but are costly to the actor, while mutually beneficial behaviours benefit both the recipient and actor.^2^ Altruistic behaviours are difficult to explain as they are costly and natural selection favours those traits which contribute to reproductive success. However, the focus of this article is mutually beneficial cooperation. Even when cooperation is mutually beneficial, it still demands an explanation. This is because if profit is differently distributed, or if coordinating action is noisy and involves costly mistakes, some agents may do better by not cooperating or may choose to avoid risks even if they would. In other words, “it is not enough for cooperation to be profitable – the distribution of profits must encourage further cooperation” (Sterelny, 2021: 53). There are two forms of potential cheating that can jeopardise this. The first is free-riding, where agents do not contribute their fair share of the effort yet still receive an incommensurate portion of the profits, increasing the cost of cooperating for other players. The second is bullying, where agents monopolise the profit.

There are many existing explanations of how mutually beneficial cooperation is sustained, including, for example, reciprocal altruism (Trivers, 1971) and indirect reciprocity (Alexander, 1987). The explanation of focus in this article is commitment. Schelling (1960) introduced commitments in his work on bargaining and conflict in game theory. Under Schelling’s formulation, commitments operate by irreversibly reducing an agent’s payoffs for a particular action or by removing options for the agent, such that it induces the other agent to choose in her favour. The alteration of the agent’s incentives could be achieved by worsening one’s payoff in the event of non-fulfilment or by delegation of control to another. Such commitments are communicated (verbally or non-verbally) to the other agent, which alters her expectations. In Schelling’s words, “the commitment is a strategic move, a move that induces the other player to choose in one’s favor. It constrains the other player’s choice by affecting his expectations… a rational second player can be constrained by his knowledge that the first player has altered his own incentive structure” (Schelling, 1960: 122-3). It is supposed that these are situations in which, by undertaking a commitment, an agent optimises her long-term utility even though the alternative option looks suboptimal in the short-term.^3^ Let us define a commitment as follows (Khan, 2024).

### Definition

A commitment is a pre-play signal in a strategic interaction taken at time t, that increases the sender’s relative payoff for carrying through option X at time t + n, and increases the receiver’s expectation that the sender carries through option X.

In other words, the commitment makes upholding the agreement profitable, by adding a cost to reneging. We can illustrate this in game theory. In a game of mutual benefit, such as a Stag Hunt (Table 1). The game involves two players who simultaneously choose to either hunt a stag or hare. Their choices are indexed 1 and 2 to represent Player 1 and Player 2. The first number in each cell represents the payoff to Player 1 of choosing that option in conjunction with the choice of Player 2. Both Players 1 and 2 would do better by cooperating and playing Stag, since they each receive of payoff of 3. However, without assurance that the other will play Stag, a player is incentivised to play Hare (to receive a payoff of 2 rather than 0, the lower payoff resulting from trying to hunt a stag alone and failing). A credible commitment to play Stag can serve to secure the mutually beneficial outcome for both agents as, by definition, it changes the sender’s incentives to play Stag and changes the receiver’s expectation that she does so. To so do would mean lowering the payoff of Hare such that Stag is now the dominant option for the player (illustrated for Player 1 in Table 2). If the receiver believes the sender will play Stag, she is motivated to play Stag, too. The same analysis will apply to games with multiple players, for example, in an *n*-player Stag Hunt or threshold public goods game.^4^

**Table 1:** The Stag Hunt

	Stag_2_	Hare_2_
Stag_1_	3,3	0,2
Hare_1_	2,0	1,1

**Table 2:** The Stag Hunt with player 1 committing to stag

	Stag_2_	Hare_2_
Stag_1_	3,3	0,2
Hare_1_	0,0	−1,1

Note that these signals need not be intrinsically costly or difficult to fake. If this is so, what are the means of ensuring the commitment is credible? Nesse (2003) distinguished four kinds of enforcement mechanisms for commitment which confer credibility to the signal. Self-enforcing commitments are secured by the fact that they make an option impossible, for example, tearing off one’s steering wheel or burning a bridge behind oneself. Contractual commitments are secured by external incentives controlled by third parties. Here, the option to renege on the commitment is still available to the agent but no longer advantageous. An example is enforcement via a lease agreement, where breaking the lease entails a financial cost. The final two types of commitments – emotional and reputational – are what Nesse refers to as “subjective” commitments. Emotional commitments are enforced by feelings such as pride and guilt. Reputational commitments are backed by the pledge of one’s reputation, for example, in taking a public oath. Frank (1988, 2011) has argued that the evolution of cooperation is at least partially dependent on the realisation of promises backed by subjective enforcement mechanisms, particularly emotion.

Elsewhere, I have argued that reputationally-enforced commitments were a crucial part of the evolution of human cooperation (Khan, 2024, 2025). In particular, I believe pre-linguistic commitments secured the stability of cooperation in early hominin group hunting as early as two million years ago. This small-scale collaboration via commitment then enabled the formation of our proto-language and the expansion of the group into a multi-level society. This resulted in the emergence of more sophisticated communication among members of the wider group, culminating in language. Linguistic commitment then allowed for more effective communication in cooperative ventures and expanded the range of cooperative activity that could be undertaken. As such, commitment and cooperation coevolved. Language allows us to commit with more specificity, to commit to spatially and temporally remote events and to make conditional commitments, among other benefits. Language and reputation sharing also increases opportunities for detection and punishment of false commitments. Both of these forms of commitment – pre-linguistic and linguistic are reputationally-enforced. Indeed, Schelling himself notes that this is a major feature of promising. He writes, “what makes many agreements enforceable is only the recognition of future opportunities for agreement that will be eliminated if mutual trust is not created and maintained, and whose value outweighs the momentary gain from cheating in the present instance. Each party must be confident that the other will not jeopardize future opportunities by destroying trust at the outset” (Schelling, 1960: 45). As such, reputationally-enforced commitments were a crucial part of securing cooperation in much of human history.

In this article, I offer a taxonomy of a new, and more effective means of commitment – institutionalised commitment. This is both contractually and reputationally enforced. I detail its advantages as well as explicate the changes in our cooperative landscape occurring in the late Pleistocene and Holocene that highlight under what conditions this form of commitment might have arisen. In particular, I focus on the transition from largely mobile foraging to more permanent settlements in the Mesolithic and an economy based on domesticated livestock and cereals, characteristic of Neolithic economies. These transitions occurred independently in a number of places, at different times. Some occurred approximately 10 kya (thousand years ago) in the Fertile Crescent and Near East, and others as late as 4 kya in areas of Northern Europe. However, importantly, with such changes, there were expansions in the scale and cost of cooperation (Sterelny, 2011). The argument I present involves some speculation, but it will generate testable predictions for understanding when and how the environmental changes underlying institutionalised commitment arise.

In Sect. 2, I discuss how institutionalised commitments work and their advantages. In Sect. 3, I suggest the demands of agricultural life and hierarchical organisation introduced a more powerful enforcement mechanism than reputation -- third-party punishment. With the formalisation of institutions, third-party punishment became a powerful and organised policing mechanism to underscore commitment and thereby further our cooperative practices. In Sect. 4, I address the problem of potential deception. In Sect. 5, I conclude.

## Institutionalised Commitment

### A Taxonomy

First, it is important to note that institutionalised commitments work in the same way as commitments based on reputation, such as promises. That is, a pre-play signal changes both the sender’s incentives for following through on a future course of action (by adding costs to reneging) and the receiver’s expectations of the sender’s intended action (Khan, 2024). The pre-play signal here may be a verbal utterance just as in the case of a promise, or it may take a different form, such as a written contract. The key difference between these two forms of commitment is the enforcement mechanism. With institutionalised commitments, the change in sender payoffs are not (only) a result of a tarnished reputation. Instead, the cost of reneging on one’s commitment include financial costs, imprisonment, or other punishments, enacted by a *third party*. These differences are important, since the greater the cost of reneging on one’s commitment, the more *credible* the commitment signal is to the receiver. While social exclusion is a low-cost form of punishment, other financially or physically punitive measures can increase the potency of potential punishment while simultaneously reducing costs for the receiver if such punishment is enforced by third-parties instead. Sometimes these costs are imposed coercively and sometimes these costs are willingly incurred by the sender. There are many scenarios in which institutionalised forms of commitment can be detrimental to human cooperation, but this will not be the focus of this article. Rather, I will be elucidating the benefits of institutionalised commitments for cooperation.

Legal commitments offer a prototypical example of an institutionalised commitment. Such contractual commitments are externally enforced. To elucidate, Sandy (the sender of the commitment signal) may enter into a lease agreement with Betty (the receiver). Here, Sandy is the tenant and Betty is the landlord. After making this commitment, Sandy’s payoffs have changed for cooperation, but the punishment for defection need not be enacted by Betty. There is a third-party punisher responsible for enforcement of the terms of the commitment – the National Residential Landlord Association or court system. Betty’s expectations of Sandy’s intended action have changed in the way we would expect of a commitment – she expects rent – but, unlike the reputationally-enforced commitments appearing earlier in our history (Khan, 2024, 2025), Betty is not the only one to whom Sandy is liable.

Using our earlier definition, a commitment is a pre-play signal in a strategic interaction taken at time t that increases the sender’s relative payoff for carrying through option *X* at time *t + n*, and increases the receiver’s expectation of the sender carrying through option *X*. Here, the pre-play signal is the undertaking of the legal contract and option *X* is paying rent. Time *t + n* indicates when the rent is due. In this particular example, the lease agreement may be a mutual commitment, with Betty offering to provide basic repair services for Sandy. Here, enforcement is conducted in an organised manner, it is common knowledge what the sanctions of violating the lease agreement are (as per the contract), and the enforcement is undertaken by a (typically) publicly-funded policy body such as the National Residential Landlord Association, the court system or Tenant’s Union.

Note there is a sense in which the undertaking of the contract serves as a precondition to the game being played, rather than a pre-play signal within the game. In fact, the contract can serve both roles. In some cases, a game will not be entered unless a contract is signed. This can be seen in, for example, an employee-employer interaction where, in order to gain the mutual benefit of pay and the service provided, a contract has to be signed. If it was not signed, the game would not be initiated. However, there are many scenarios in which a contract can be written after the game is already specified. It is easy to think of many scenarios in an office where a written plan of action is agreed upon once we are already aware of the project that needs to be completed; when we are already “playing the game” as such. Indeed, it is important to note that even when contracts are a pre-condition to playing the game, they still fulfil the same function of the “pre-play” signal in the provided definition (Khan, 2024). That is, contracts change sender incentives and receiver expectations in just the way we would expect of a commitment signal. The only difference is that this signal may also determine whether or not the game is played at all.

So let us say that a prototypical institutionalised commitment is one where the third-party punishment for reneging on a commitment is conducted in an organised manner, it is common knowledge that transgressive behaviours will be punished, and the punishment is carried out by a publicly-funded policing body, illustrated by many modern legal commitments. These contractually-enforced commitments allow us to cooperate with strangers with reduced risk of defection and therefore facilitate the spread of cooperative behaviours. However, institutionalised commitment is not an all-or-nothing matter, and instead comes in degrees. In this section, I lay out a basic taxonomy that explicates the key features of institutionalised enforcement. Indeed, it is impossible to provide a catch-all definition of institutionalised commitment because the way it has been actualised in different cultures differs so drastically. No detailed account of what a policing body is in one culture will apply perfectly to another. Instead, let us delineate three different forms of institutionalised commitment, call these “minimally institutionalised”, “partially institutionalised” and “fully institutionalised” and elucidate their defining features.

**Fig. 1:**
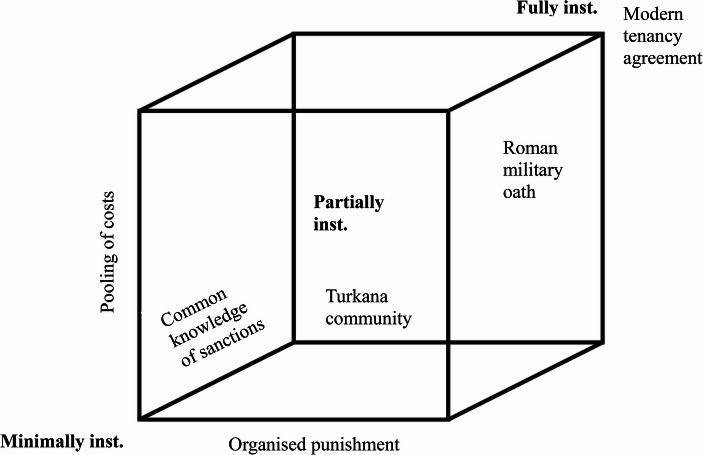
Diagrammatic depiction of types of institutionalised commitment, based on enforcement enacted by third parties

In the most minimal case, we are defining institutionalised commitments as those which involve punishment by third parties, regardless of whether this punishment is organised, is commonly known or involves a pooling of resources. Yet, as noted earlier, many of the canonical forms of institutionalised commitment we see today involve third-party punishment in a much richer way. There is also a middle ground between “minimally institutionalised” and the “fully institutionalised” commitment exemplified by the modern tenancy agreement. A “partially institutionalised” commitment can be one with only some of the features illustrated in Fig. 1, or one possessing all of these features, but to varying degrees. For example, there might be organised punishment to some degree but with only minimal or no pooling of costs. In this case, we would not have a publicly-funded policy body as we do in the case of a fully institutionalised commitment. Or, there might not be common knowledge about what sanctions are entailed in failing to meet the commitment, but yet punishment is carried out by organised third parties, the exact consequences unbeknownst to the committer. What might such punishment structures look like?

Mathew and Boyd (2011) illustrate how community-imposed sanctions for cowardice and desertions in war maintain cooperation in the Turkana community, a politically uncentralised, egalitarian, nomadic pastoral society in East Africa (Mathew & Boyd, 2011). Here, there is no designated body which carries out a policing role, yet enforcement is partially institutionalised in that punishment is conducted in a somewhat organised manner under conditions of common knowledge, and the cost for enforcing these norms fall on other members of the community. At least one person was sanctioned in 47 per cent of raids where desertion was reported and in 67 per cent of raids where cowardice was reported. Desertion and cowardice are forms of reneging on the commitment to participate fully in the raid. Acccusations of defection require consensus from the group and punishment is carried out by third parties – members of the violators age-group – even if they did not participate in the raid and did not experience the consequences of the violation. Serious cases involve not only verbal sanctions but severe corporal punishment, where the defector is tied to a tree and beaten by his age-mates. Note also that the punishment here is based on cultural norms about the wrongness of such a transgression, in the sense described earlier, rather than for the gain of the punisher.

Another example is the sacramentum militare (also known as militum or militiae), the oath taken by soldiers in pledging their loyalty to the emperor in the Republic era onwards. The oath took the form: *Iurant autem milites omnia se strenue facturos quae praeceperit imperator*,* numquam deserturos militiam nec mortem recusaturos pro Romana republica!* (“But the soldiers swear that they shall faithfully execute all that the Emperor commands, that they shall never desert the service, and that they shall not seek to avoid death for the Roman republic!”). If the soldier reneged on this commitment, he was subject to harsh penalties, such as execution or corporal punishment enacted by a policing body.

Note also that it is not simply punishment which is doing the work to sustain cooperation here. Commitment allows us to achieve something over and above cooperation ensured by punishment alone – that is, it involves a pre-play signal (for example, an oath or contract) which allows the agent to identify cooperative others and choose to preferentially interact with them. That is, it allows for effective partner choice. In this way, commitment allows for cooperation without requiring us to resort to punishment – although the threat of punishment is what makes the signal credible. This contingent cost is what makes the receiver believe that the sender’s incentives have changed. Indeed, as Schelling writes, “if I have every reason to sue for damages in the event you do me harm, saying so is not a threat, it is merely a communication, what I call a warning. Arranging incentives so that I must sue whether I want to or not and communicating that I must do so is the threat” (Schelling, 2003: 52). Previously, these punishment costs might have come from opening up oneself to reputational consequences. In the case of institutionalised commitment, these punishment costs also come in the form of physical and financial penalties enforced by third parties.

### Advantages of Institutionalised Commitment

What fitness benefit do these institutionalised commitments afford us at the level of sender and receiver, over and above other, non-institutionalised forms of commitment? First, in legal commitments, the terms of a promise are typically made clearer than in our previous forms of commitment such as pre-linguistic and linguistic commitment (Khan, 2024). While, of course, a non-legal commitment can also be written and a legal contract could be oral or poorly defined, legal commitments are typically more precise. This translates into increased expectations of cooperation conditional on the signal, since the signal is more reliable concerning what counts as cooperation. If the receiver is more confident that the sender will cooperate or about how they will cooperate, she is incentivised to cooperate herself if the game is well modelled by a Stag Hunt or repeated Prisoner’s Dilemma with a cost to defection.

Second, institutionalisation aids not only partner choice but also partner control. With institutionalisation, an agent can also be forcibly made to follow through on their commitment, rather than simply punished for not doing so. In some legal agreements, parties can mandate receipt of an item in exchange for the service -- if the agreement is violated, the law can intervene to repossess the item and provide it to the receiver. If this is so, the enforcement takes the form not of a penalty but of reparations such that the commitment is forcibly followed through. Alternatively, if the good is not provided, we can be assured that appropriate compensation is received for a reneged commitment via debit or credit card chargeback. This differs from non-institutionalised linguistic commitments (Khan, 2024, 2025). Partner choice based on commitment does not represent as crucial a role since partner control can be ensured by a third-party.^5^ In such cases, agents do not need extensive information about the reputation of a new partner in order to consider it worthwhile to interact with her. As long as amends can be forcibly made, an agent’s cooperativeness is institutionally protected. One does not need to trust that the seller is reliable since one is safeguarded. Since commitments can be made to be forcibly followed through by means of third-party intervention, the cognitive demands of reputation-gathering are lowered.^6^

Third, with institutionalised commitments, the value of enacting punishment for the receiver is lowered relative to the cost imposed on the reneger. Exclusion is typically a low-cost form of punishment but enacting further punishment in terms of financial penalty, imprisonment or restitution would be very costly for an individual agent to undertake. In cases of fully or partially institutionalised commitment, however, this is enacted by a policing body. Contributions to the upholding of such a third-party policing mechanism are small compared to the difficulty an individual agent may go through to acquire her promised goods or punish the reneger. Importantly, the cost of reneging increases for the sender, disincentivising acting in contradiction to one’s commitment. The cost now includes not only reputational consequences, but physical and financial ones, too. This is perhaps the most fundamental change to our practice of making and keeping commitments – more costly forms of punishment become cheaper and less risky, increasing our means of making commitments credible.

Now that we have understood what institutionalised commitments are, how might such forms of commitment have emerged in our evolutionary history? Below, I offer an account. Of course, as noted earlier, there are many different kinds of third-party punishment and institutionalised commitments, with different explanations for their origin. However, I argue that changes in our cooperative landscape (the kinds of problems we faced) and a change in our socio-organisational structure would have been precursors to the emergence of institutionalised commitment in general. One key change was a shift from foraging lifestyles to agriculturally-focused market economies. Another is the shift from egalitarian to hierarchical societies. Such changes, though they need not be the only ones, played a critical role in laying the foundation for a new means of enforcement for commitments – institutionalised third-party punishment. The claim is not that institutionalised commitment arose at the same time as these changes to our socio-economic structure but that such changes in our cooperative environment were pivotal to the emergence of institutionalised commitment – that is, they provided the right kind of *starting conditions* for the emergence of this form of commitment, and the *further resources* to increase the effectiveness of such commitments.

## Changing Cooperative Landscapes Arising in the Holocene

### Starting Conditions: Agriculture, Private Property and Third-party Mediation

In communities with highly interconnected social networks, commitments can be enforced via reputational consequences (Khan, 2024). However, once we have centralised states and political leaders approximately 5 kya, we see the omnipresence of third-party punishment of agreements backed by coercive force (Pinker, 2011; Boehm, 2012). By the Common Era, we had the kinds of societies we are familiar with today, with large cities, bureaucracies, taxes and formal punishment structures (Gowdy, 2021). In order to understand how we got here, we first need to understand the forces driving the development of personal entitlement and power which led to the emergence of this new means of enforcement – third-party punishment. These were features that were relatively uncommon in early hunter-gatherer communities. The purpose of this section is not to explain the emergence third-party punishment simpliciter. Explaining the evolution of third-party punishment simpliciter is a much larger task than can be accomplished in this article.^7^ The target of the current section is to explicate some important relationships between the rise of agriculture and property rights enforced by mediators (Sect. 3.1) hierarchical societies and the stabilisation of third-party punishment (Sect. 3.2) and institutionalisation (Sect. 3.3), which led to the development of a new and more effective means of commitment.

While, in the volatile climatic conditions of the late Pleistocene, sharing would be more pronounced, at the turn of the Holocene, climate variability had reduced. Reduced variability opened avenues for farming, private property and inequality (Bowles & Choi, 2013; Richerson et al., 2001; Mattison et al., 2016). Richerson et al. (2001) go so far as to say that agriculture was not possible under the last-glacial conditions due to high-amplitude fluctuations in climate, but became mandatory in the Holocene due to climate stability and intergroup competition in which those groups who developed more intensive subsistence strategies would have prevailed. These changes have often been attributed to the “Neolithic revolution”, a term invented by Gordan Childe in 1932 to characterise the domestication of wild plants and animals starting in Mesopotamia. However, the Neolithic revolution occurred in different places in different times, including the Near East, China, sub-Saharan Africa, Mesoamerica, and South America, with different kinds of plants being domesticated in different regions. Rather than offering a comprehensive account of when and where such changes emerged, we will discuss how, in general, they altered our cooperative landscape.

Agriculture introduces new problems for cooperation – risk management and the rise of inequality. In relation to the first, farming introduces a long gap between investment (land preparation and crop planting) and the subsequent profit (Winterhalder & Kennett, 2009). The investment is risky as the weather, other humans, or other animals can jeopardise the profits of the investment during that period. As noted by Sterelny (2011), these risks are multiplied by the reduction in mobility, the shift to reliance on few resources and on storage – which is, itself, risky in the introduction of spoilage. Many Neolithic economies depended on surplus production of grains and a complex division of labour compared to simple hunter-gatherer communities. This kind of economy often, but not always, encourages the development of private property rights that entail sanctions if violated (Bowles & Choi, 2013; Gintis, 2007). If not, individuals leave themselves open to free-riding. This is well illustrated by the case of the members of the Batek community in Malaysia who discovered cultivated rice. They planted some, however, their crop was harvested by others and simply shared among the group (Endicott, 1988). Similar cases of free-riding are found in the first farming attempts of in the! Kung community in southern Africa and the Hiwi in Venezuela (Bowles & Choi, 2013; Wiessner, 1982).

Of course, there are exceptions to this purported relationship between agriculture and private property rights. Ostrom (1990) documents some societies in which common pool resources are managed without need for private property rights or centralised control. In the Near East, along the Indus river, coastal China, and the Valley of Mexico, early agricultural societies (10–12 kya) were based in wetlands making agricultural development fairly easy and supplementing this with access to fish, aquatic plants and animals (Gowdy, 2021).

Bowles and Choi (2013) note two conditions for private property of dwellings, animals and crops to persist – that most people accept the exclusion of others from one’s possessions as legitimate and that things which we were valuable could be made possessions, i.e. unambiguously demarcated and defended. These norms do not rely on complex institutions but rather shared sets of beliefs and expectations of conformity and so plausibly represent an early emerging form of informal institutionalisation. Indeed, Gintis (2007) shows how a respect for private property can emerge in the absence of legal institutions ensuring third-party enforcement. This is modelled as an endowment effect in a Hawk, Dove, Bourgeois game.^8^ The model shows the existence and stability of a decentralised, self-enforcing type of private property based on behavioural propensities. In fact, we even see these kinds of propensities to enforce private property in the territorial behaviour of many animals and in primate communities (Torii, 1974; Sigg & Falett, 1985). So, the predisposition to respect private property in humans predates modern civilisations and judicial institution. For Gintis, it relies on a form of loss aversion. That is, the incumbent is willing to commit more resources to defending her property, *ceteris paribus*, than an intruder is willing to commit to taking the property from her. Arguably, this kind of respect for property lays the basis for more institutionalised rights (Gintis, 2007). Importantly, with private rights comes *sanctions* for violations of these rights.

As mentioned earlier, whilst many foraging societies resolved disputes via individual punishment in the form of social ostracism, the stakes became higher in the more settled, agricultural communities of the Neolithic. Fry (2000) discusses how third-party mediation was involved in sedentary tribes. Here, band members may try actively to distract the participants, facilitate negotiations, or otherwise serve as mediators in conflicts between two individuals. Modern ethnographic equivalents can be seen among the among East Indians of Fiji (Brenneis, 1990), the Limbus of Nepal (Caplan, 1995), and the Abkhazians of the Caucasus (Garb, 1996) where conflict resolution can involve mediation from one or more elders. This is also seen in the Dou Donggo community of Indonesia where there is also the option of appeal to the village headman or adjudication in the court systems (Just, 1991). Examples of adjudication where a judge not only offers a verdict but can also enforce it are seen among the Fiji Islanders (Arno, 1979) and the Tarahumara of Mexico (Pastron, 1974).^9^ Boehm (2012) similarly notes that where there were more sedentary tribes, avoidance became too costly an option, encouraging farmers to allot authority to chosen leaders as conflict mediators.

Of course, some foraging communities may also have involved third-party mediation. Notably, the Yurok and Hupa hunter-gatherer communities of Northern California in the 19th century and the Karok community of Oregon, involved third-party mediation in disputes by a legally knowledgeable “judge” (Kroeber, 1925; Boyd & Richerson, 2022). An important principle of their legal system, however, was the recognition of private property, where rights and possessions were individual, rather than collective (Boyd & Richerson, 2022). Again, this suggests that the establishment of private property promotes an important change in our resolution and punishment practices, setting the stage for the development of third-party punishment.

But why mediate if mediation is itself costly? This is known as the second-order problem of altruism -- punishing non-cooperators is itself costly, so punishment by third parties is also in need of explanation (Boyd et al. 2003). It is worth noting that in small groups, the collective benefit created by sanctioning may be enough to compensate the individual who enacts the sanctioning (Boyd & Richerson, 2022; Singh et al., 2017). Indeed, Sasaki and Uchida (2013) note that exclusion involves a natural benefit to the agent because exclusion decreases the number of beneficiaries of a public good. As long as the cost of driving the free-rider away is less than the reallocated benefit, exclusion will be incentivised. However, in larger groups, it no longer pays to punish as the effect of a defector on any particular individual reduces.

There are a few possible explanations for the stability of sanctioning in larger groups. Perhaps onlookers are engaged in a different strategic interaction. The game that the onlookers are playing may involve signalling their virtues through enacting costly punishment. Jordan and colleagues (2016) develop a model in which they show that enacting costly third-party punishment can serve as just such a signal of trustworthiness to others. They empirically validate this model by showing that third-party punishers are trusted more in experimental setups where they punish a “helper” for an insufficient contribution to a recipient in a Dictator Game. This increased trust is demonstrated by a subsequent Trust game played with the punisher, where others are more likely to trust them after they have engaged in third-party punishment. It was found that agents sent 16% points more of their endowment to third-party punishers than non-punishers. The punishers themselves also behave in a more trustworthy manner in the subsequent game, showing that this increased trust in them is warranted. It was found that those who punish return 8% points more of the entrusted endowment to the original agent than those who do not punish. If punishment can act as a costly signal of one’s trustworthiness within one’s subgroup, this may be and explanation of why third parties enforce agreements in dyads even when their interests will not be directly harmed by a foregone agreement. Such costly signalling has been modelled by Gintis et al. (2001).

Relatedly, conformist transmission has also been shown to solve the second-order problem of altruism and incentivise punishment, and is not unlikely conformism was present in societies of this time (Henrich & Boyd, 2001). I will return to the second-order problem of altruism again in the next section. The important point is that with the more stable climates of the Holocene, we saw the emergence of agricultural societies in many different parts of the world and, often, the development of agriculture was strongly connected to sedentism, the development of private property rights, and third-party mediation of conflict. This is not to say that it is the *only* important factor in the rise of third-party mediation – just that it likely had a significant role to play.

### Middling Conditions: Inequality, Hierarchy and Stabilisation of Third-party Punishment

Agriculture is often believed to be connected to the rise of inequality, which has important consequences for the stabilisation of third-party punishment. Indeed, Hooper et al. (2018: 10) argue that “intensive agriculture transformed the economic value of territorial resources, and created the crucible that produced the chiefdoms and states characteristic of the middle and later Holocene. Sedentism and higher population densities may have also reduced the costs of alliance formation, enforcement, and cooperation in ways that reinforced these outcomes. Mobile foragers relying on widely or unpredictably distributed plant and animal resources, on the other hand, have been less likely to go down this road to increasing sociopolitical complexity.” However, while large-scale, highly stratified state systems are highly correlated with the emergence of intensive agriculture, as noted earlier, there are also case in which agricultural societies lack such institutions, as well as hunter-gatherer societies that demonstrate high degrees of political and economic inequality, hereditary inequality, and enslaved labour, along with sedentism and political complexity (Bowles & Fochesato, 2024; Smith & Codding, 2021; Boyd & Richerson, 2022).^10^

While the highest rates of intergenerational wealth transmission and inequality occur in agricultural societies where rights to resources are often permanent, the true relationship between agriculture and rise of inequality is complicated and varies by region (Mattison et al., 2016).^11^ There are many potential causes of inequality and complicated interaction effects between these causes. To name a few: the advent of defensible natural resources (based on density and predictability) such as fish captured in weirs, or the highly clumped distribution of acorns in the Northwest Coast of California that allows for territorial ownership; material wealth transmission usually in the form of agricultural land; control of production technology; intergroup conflict and the need for elites to manage conflict; population-pressure that introduces competition during resource scarcity; population density and the need for elites to resolve coordination difficulties; and the influence of prestige “aggrandizers” (Mattison et al., 2016; Smith & Codding, 2021). It is not my purpose in this article to adjudicate on these theories but rather to use pieces of them to supplement a narrative outlining what I take to be the key features in the emergence of institutionalised commitment.

Mattison et al. (2016) and Smith and Codding (2021) argue that economic defensibility of resources and transmissibility of wealth are the key factors in the emergence of persistent inequality. In particular, *clumped* (mainly aquatic) resources contribute to evolution of institutional hierarchy as opposed to population pressure, warfare or agriculture alone.^12^ These clumped resources are more likely to repay the costs of territorial defence because the area one has to defend is smaller relative to the gain (Mattison et al., 2016). They also allow some individuals or factions (e.g. kin groups) to extract benefits from subordinates such as labour and political allegiances in the form of “patron-client” systems. These “patron-client” systems ensure outside options are less attractive than subordination to patrons (Smith & Codding, 2021).^13^ Empirically, the Native American populations of the northern Pacific coast show a correlation between prime fishing sites and social inequality, and irrigated farming is associated with a stratified class structure (Smith & Codding, 2021; Braudel, 1972). Prentiss et al. (2012, 2014) also show how prehistoric salmon-fishing villages in British Columbia responded to resource competition with households tightening their control of key resource sites and allowing others to reside as subordinates.

It is important not only to understand when and how inequality began, but also under what conditions it *persist*s. Bowles and Fochesato (2024) note that while material inequality likely appeared in western Eurasia at the end of the Paleolithic approximately 12 kya, it tends to show up sporadically and does not persist prior to the Bronze age (3-1.5.5.5 kya).^14^ Bowles and Fochesato (2024) suggest that the “aggressively egalitarian” (Hodder, 2014) nature of some Neolithic communities mitigated the spread of inequality even after the advent of agriculture, modelling the coevolution of wealth inequalities with collectivist social norms in an agent-based simulation (Bowles & Fochesato, 2024). In the late Neolithic and especially from the Bronze Age and onwards, inequality became self-reproducing. They argue three changes led to enduring inequality long after the agriculture: (i) technology; (ii) institutions; and (iii) culture.

When forager societies began to rely more heavily on resources such as wild grains and seeds as a result of more stable climates, which are amenable to storage and surplus production, there was a crucial shift in our potential to accumulate wealth (Richerson et al., 2001). Storage-ready crops allow successful households to disengage from the risk-mitigation strategies of a community because they allow for private accumulation rather than egalitarian redistribution (Bowles & Fochesato, 2024). This was furthered by technological changes in the late Neolithic. Ox-drawn plows raised the value of land, draft animals and other forms of material wealth relative to human labour, concentrating wealth in a more select few (Bowles & Fochesato, 2024). Farming became more land-and capital-intensive and this increased the benefit to those who already controlled said resources. Greater inequality as a result of plow-based cultivation as well as storage-ready crops such as cereals is well documented (Alesina et al. 2013; Boserup, 1965; Goody, 1976; Testart, 1982).

As we have noted earlier, there is an association between clumped resources and material inequality. This association is strengthened in the presence of technologies for transport from resource sites, such as watercraft, and better means of harvesting of clumped resources, such as scythes (Mattison et al., 2016). As such, technological changes to agricultural practices would have had effects on the persistence of material inequality by allowing clumped resources to be better utilised. Not only this, but certain technological innovations have been strongly associated with monopolisation – for example, among the Chumash of the central California coast, hereditary chiefs financed and controlled planked canoes (Arnold, 2010).

With such changes, stronger property rights and norms of respecting such rights arise (Mattison et al., 2016). When coupled with institutionalised means of protecting these resources, this inequality becomes persistent (Mattison et al., 2016). Indeed, Bowles and Fochesato (2024) argue that one of the key factors in the development of proto-states was the changes in the domain of activity governed by private property rights. Gowdy (2021) notes the first well-documented large-scale culture was Sumer in what is now Mesopotamia. It was composed of several villages in approximately 7 kya and, by 5 kya, much of the land was owned by family groups who controlled “temple estates” managed by the rulers. In Bronze-age Mesopotamia, we saw more of a kin-ordered, decentralised form of political rule (Stasavage, 2020). Such proto-states were characterised by an increase in the elite power, providing the political and economic conditions for inequalities to be perpetuated (Bowles & Fochesato, 2024).

A final important development in the rise of inequality and the change in our cooperative landscape was culturally constituted. Graeber and Wengrow (2021) argue that the origin of economic inequality proper lies in the emergence of kings, priests, overseers and judges – such inequality is not the result of agricultural changes but political and institutional changes. For Graeber and Wengrow (2021), the cause is cultural. That social norms sustain inequality is also supported by a number of models in evolutionary biology.^15^ Of particular importance is religion. Religion is a multifaceted phenomenon comprising of many distinct behavioural practices, such as belief in the supernatural, norms concerning appropriate behaviour, myths, ritual and more (Lindenfors & Svensson, 2021). Some argue religion evolved as a by-product of other selection for other traits and others argue that it has fitness benefits, for example, in group cohesion. I will not attempt to give an evolutionary account of its origins here, but rather draw our attention to the fact that religion can play a role in the rise of inequality and the strengthening of third-party punishment – crucial features of institutionalised commitment.

Sterelny (2021) offers a lengthy discussion of how organisation based on prestige arises in cases where there is special access to ritual or esoteric knowledge, as well as how religion can legitimate new forms of inequality.^16^ Indeed, many religions promote structured organisation on the basis of authoritative ranks, such as priests or, less formally, religious teachers. At the same time as changing our cooperative landscape in ways that demand new means to ensure our interests our safeguarded, these changes give us even more means of enforcing commitment. They connect the fulfilment of those commitments to normative standards possessed by the group as a whole, making them targets for third-party punishment.^17^ Indeed, modern psychological studies have also shown that religious priming promotes costly punishment of unfair others in economic games – the sort of punishment typically required of institutionalised commitment (McKay et al., 2011). Experimenters suggest this may be due to two effects: the first is the worry that a supernatural being will punish non-punishers since they have not enforced norms themselves. The second is that religion primes us to think of cultural norms of fairness and its enforcement. Religious priming has also been shown to reduce cheating (Randolph-Seng & Neilson 2007).

It is important to note that the demands of cooperation increase when there is greater inequality, an increase in community size, a decline in intimacy and delayed return on investments (Sterelny, 2019). Of course, we should not equate all foraging communities with small-scale societies and agricultural economies with large-scale societies. Some late Pleistocene and Holocene hunter-gatherer communities had large social networks and engaged in large-scale cooperation, including in the creation of drivelines, hunting nets and fish weirs, as well as in other realms such as warfare, alliances, trade networks and habitat modifications such as burning and irrigation (Boyd & Richerson, 2022; Singh & Glowacki, 2022; Bird et al., 2019; Hill et al. 2014). Nonetheless, we do see a general decline in egalitarianism, the development of hierarchical structures and delayed return on investment resulting from these socioeconomic changes in the Neolithic, and this would increase the importance of developing a new enforcement mechanism to ensure commitments are followed through. Rather than relying only on reputational consequences, if the practice of third-party mediation is already at play, this affords us with an enforcement mechanism which can be used to secure commitment in the more complicated collective action problems we then faced.

In some places, there are gaps of thousands of years between the establishment of agricultural societies and political states (Sterelny, 2011). It is plausible that incremental changes toward a more agriculturally intensive, sedentary lifestyle also incrementally favoured increased organisation, frequency and strength of punishment as our cooperative enterprises and hierarchical structures developed. Chiefdoms may represent a transitionary step between villager societies and larger political units, and these involved an intermediate increase in the power of elites to enforce punishment. Indeed, in the kind of transegalitarian societies that preceded formal institutionalised states, local leaders served a mediating role in conflicts often based on prestige derived from skill and expertise. These were societies in which political leadership goes beyond traditional kinship relations but that lack clear politically centralised or institutionalised forms of power. Transegalitarian leaders are termed by Sahlins (1963) “Big Men”, and are particularly well-documented in Melanesia (Sahlins, 1963).

Crucially, as with religion, the rise of elites is important to solving the second-order problem of altruism and thereby stabilising third-party punishment. Hooper et al. (2010) show, using a repeated public goods game, that there are circumstances in which agents might do better living under the rule of a leader, i.e. if paying a “tax” to the leader to monitor and punish non-cooperation and non-tax-paying outperforms failing to cooperate at all or cooperating in smaller, leaderless groups. For leaders, enforcement can become individually profitable, thereby solving a second-order problem of altruism.^18^ Likewise, Ozono and colleagues (2016) show how, in public goods games, leaders in a group can stabilise cooperation by punishing both non-cooperators and non-supporters (those who do not punish other non-cooperators) and they in fact benefit when this is the case, favouring the evolution of punishment and second-order punishment. Supporters will be incentivised to punish renegers of commitment if they will incur a cost for non-punishment and if their punishment signals trustworthiness to others. It is also possible the cost of punishment is lower for group leaders, since they are most likely to get support and will not therefore be the sole target of potential retaliation.

So, I have suggested that agricultural life provided the breeding ground for private property rights where disputes are mediated by third parties. Third-party enforcement of such agreements may have arisen among agents interested in signalling their trustworthiness to others within their subgroup. The transition to more unequal, hierarchically organised societies would have made an effective enforcement mechanism such as third-party punishment even more important given the complexity of interactions and would have added further resources for corralling support. However, we have not yet addressed the institutionalisation of such punishment.

### End Point: Institutional Structures

Importantly, the centralisation of power and effective use of coercion limits external challenges to authority, and further ratchets up our means to contractually enforce commitments. Bowles and Fochesato (2024) cite the Roman Empire arising approximately 1.5 kya is especially indicative of enduring inequality, representing a unified elite class and a monopolisation of resources. Such dramatic changes in hierarchy and power changed our cooperative environment in ways that would set the stage for powerful political institutions which could act as enforcers of commitments. With the development of community bodies which uphold norms concerning interaction, third-party punishment has scope to become institutionalised, and as we develop more precise structures regulating interaction, third-party punishment become more powerful. Eventually, we arrive at modern governments and courts which can enact third-party punishment on behalf of agents where commitments are not upheld. Where might this institutionalisation have come from?

First, it is important to note that there is a difference between informal and formal institutions. This difference is on a continuum and spans multiple dimensions. Generally, formal institutions are characterised by constitutions, contracts, and forms of government (Kaufmann et al., 2018), whilst informal institutions will include “traditions, customs, moral values, religious beliefs, and all other norms of behavior that have passed the test of time” (Pejovich, 1999: 166). More precisely, while informal institutions typically govern personal exchange, are unwritten, non-contractual, are premised on shared expectations and are enforced privately, formal institutions govern impersonal exchange relations (for example, trade), involve written, contractual rules of law, are premised on organisational goals (for example, societal order), and involve third-party enforcement (Hyden, 2006). Of course, institutions can be more or less formal depending on whether they have some or all of these features and the sophistication of these features. For example, Australian aboriginals have complex kinship systems accompanied by explicit norms. This institution will be more formal than one where the norms of interaction are not explicit, even though it does not involve impersonal exchange relations. So an institution may be more or less formal in one of the relevant aspects of formality without being so in another. Furthermore, though there may be instances of formal institutions which do not involve third-party punishment; all that will be relevant to our current discussion will involve third-party punishment.

One might already note that informal institutions would have been present in early hunter-gatherer societies in the form of obligations in kinship relations and food-sharing rules. These informal institutions would have aided in the transition to Neolithic agricultural economies and the development of more formal institutions by creating shared expectations of cooperation on which more complex interaction could be based. However, the more complex the interaction, the greater the need for formalisation to safeguard cooperation, since institutions make mutual expectations explicit, reducing ambiguity and transaction costs. It is very likely that institutions were becoming increasingly formal just as third-party punishment was becoming even more important for securing cooperation – war and private property rights represent paradigm examples of this relationship (Powers et al., 2016). Of course, not all institutions appear incrementally and not all appear to safeguard cooperation. We are interested in the subset of institutional structures that do.

Formal institutions offer a number of advantages. They can lead to more efficient cooperative outcomes (Powers et al., 2016). First, where individuals are subject to time-discounting and may choose irrationally as a result, institutions can set up incentive structures that promote optimal long-run choices. Second, where information about others in one’s group is not readily available given growth of the group size, institutions or institutional membership may provide such information. Indeed, the smooth functioning of a market or any large-scale enterprise is hindered by incomplete information (Hayek, 1945). However, institutional rules that enforce information flow help us to achieve these rewards (Binmore, 2005). Third, in relation to agriculture in particular, groups with institutional rules regulating irrigation have been demonstrated to successfully solve collective action problems (Powers & Lehmann, 2013). Institutional rules also regulated trade of staple goods during the Neolithic (Oka & Kusimba, 2008). These rules could have contributed to the development of larger polities as it pays to interact with others who play by the same institutional rules (Powers et al., 2016). Finally, and most importantly, the development of formal institutions comes to shape the role of third-party punishment in all of the ways suggested in Sect. 2.

Of course, this is not to say formal institutions cannot be harmful. It would be impossible to weigh up the relative harm and benefit to provide a selectionist argument for institutionalisation, but this is not my aim. I am not intending to argue that institutionalisation was selected for because of its beneficial effect on cooperation or commitment. Rather, third-party punishment and institutionalisation arose for a variety of reasons and, I have suggested some of the ways in which we might understand their emergence in history in order to understand the emergence of this new and effective form of commitment.

## The Problem of Deception

As with any commitment, we face the possibility of cheaters who signal that they will cooperate and subsequently defect. Too many cheaters can undermine a signalling system such that signals are no longer believable. How do we ensure commitments are credible in light of this? The problem of deception can be mitigated by two means: increasing the probability of *detection* of defection, or increasing the *cost* of defection.

Of course, detection of deception will depend on the relative complexity of cooperative interactions. There would most likely be a trade-off between the benefits of institutionalising commitment and the costs of increasing complexity in human social worlds. The lease agreement is a relatively simple interaction so detecting defection is easy. Given the clear terms of the commitment, the sender’s perceived probability of defection being detected increases. For example, suppose Sandy’s lease agreement specifies that she pays rent on the first day of each month. Knowing this, Sandy’s perceived probability of her defection being detected should increase as the second day of the month arrives. This is in contrast to the linguistic commitments which may be more vague – for example, commitments of the form “I will help you”. It is likely the requirements of aid in these kinds of interactions were not explicitly codified and thus whether a particular behaviour counted as defection was uncertain.^19^

So, some institutionalised commitments have the advantage that they are written down or codified more precisely than our previous forms of commitment, making the problem of deception less potent by codifying what counts as cooperation or defection and thereby keeping deception within tolerable limits. However, in more complex interactions, the explicitness of institutionalised commitments will aid in detecting defection, but it will likely not exceed the ability of detecting defection in small societies where direct observation of one’s defection is possible in shared activities. Where detection probability is lower, the pull of deception can be lowered if the cost of punishment is known and is great. Indeed, this is precisely what institutionalisation of third-party punishment does for us; increase the cost of reneging by adding financial and physical punishment.

Relatedly, third-party policing can provide us with means of preventing defection due to community oversight. In modern society, this is carried out by regulatory bodies. Consider, for example, the unilateral contracts involved in insurance policies. Not only do we have institutions offering promises in this case, but the promise is itself policed by a regulatory institution which ensures that such promises are upheld and penalises the insurance company if they are not. The Financial Conduct Authority in the United Kingdom makes sure consumers are treated fairly in insurance transactions. The Prudential Regulation Authority ensures that insurance companies have enough capital to fulfil their promises by preventative capital risk management. So potential deception may be limited by prudential oversight. This works by reducing the payoff for defection – it is risky in the face of regulatory backlash, increasing *both* the means of detection of defection and the associated cost.

Occasionally, institutionalised commitments will also involve a different kind of cost which limits potential deception. While, earlier, commitments were made credible by socially imposed costs, credibility in the case of some institutionalised commitments – notably modern legal contracts – can be enhanced by the cost of the signal itself. In order to make sense of this, let us first make a distinction between two kinds of signalling costs. There are upfront costs undertaken by the sender which self-select for honesty (Bliege Bird & Smith, 2005). Here, either dishonest agents cannot afford to signal because the cost is intrinsic to the signal itself, or it could be that the one cannot produce the signal in the absence of some underlying state – in other words, these signals are “hard to fake” (Frank, 1988).^20^ Some examples of such intrinsically costly signalling in human behaviour are feasts hosted by village leaders, gift-giving, and displays of courage in intercommunal conflict. There are also contingent costs – costs that are incurred contingent upon defection. The reputational and contractual punishment we have discussed so far are examples of contingent costs – they are enacted only if one defects.

In some kinds of legal commitments, we see upfront, intrinsic costs in addition to the familiar social, contingent costs discussed earlier. These upfront, intrinsic costs are undertaken at the making of the commitment, regardless of whether one cooperates or defects, and are imposed by the nature of the commitment itself rather than by the another party. The intrinsic cost is what serves to keep commitment signals credible, since only honest agents will signal. To illustrate, consider hiring a lawyer to draft an agreement between two individuals. The legal fees involved in hiring the lawyer serve as a costly signal which renders the commitment more credible. It signals to another agent that one intends to cooperate by the terms of the agreement and that one expects others to do so, too. An agent would be disincentivised to pay an upfront cost if the intention was to defect and it was sufficiently likely she would be caught. This is not to say one would never undergo such cost – the profit of successful defection may be great – only that upfront costs act to deter such defection in the same way that social contingent costs do.

Not all institutionalised commitments will involve such upfront costs – this is not a function of third-party policing but a function of the cultural background surrounding such institutions and the way in which they function. Nonetheless, even in the absence of intrinsic signalling costs, deception will be mitigated since a clear codification of the terms of the agreement can make instances of defection easier to *detect*, third-party punishment increases the *costs* of defection, and regulatory institutions can serve to do both. Some of these deception-mitigating mechanisms were unavailable in the case of earlier, pre-linguistic and linguistic commitments and others were less powerful.

## Conclusion

In this article, I have offered a taxonomy of institutionalised forms of commitment, their advantages, and their emergence. Institutionalised commitments prototypically involve third-party punishment for reneging on a commitment, conducted in an organised manner, where it is common knowledge that transgressive behaviours will be punished, and the punishment is carried out by a publicly-funded policing body. However, there are many different forms of institutionalised commitment and they will possess one or more of these features to varying degrees. Such commitments offer a number of fitness advantages over those commitments which are not institutionalised. They are often clearer in their terms, there are means by which agents can be forcibly made to follow through on a commitment, and the cost of enacting punishment is lowered relative to its greater deterrent effect. Institutionalised commitments allow us to make commitments with strangers where reputational information about their past behaviour is irrelevant. This is because if, for example, restitution of property is specified in the contract, we often have little to lose by entering into a commitment and facing potential defection – the court system will ensure that the receiver is paid her due. Thus, laws allow us to undertake risky promises with little opportunity for exploitation, extending the scope of our cooperative interactions.

I have argued that institutionalised commitments might have emerged with changes in our cooperative landscape in the Neolithic. Increasingly settled, agricultural life altered our cooperative landscape in ways that called for a better means of enforcement for agreements. Increasing organisation and strength of third-party punishment would have been more important as cooperation became, in general, more complex with delayed reciprocation over time. Furthermore, institutions were becoming increasingly formalised in the Neolithic, and this had implications for our practices of third-party punishment. In particular, it became organised, lower cost, and common knowledge. In hierarchical societies where a leader can corral support among other members of the tribe, punishment becomes less costly relative to the resources of elites, and more organised. With the modern policing bodies of governments and courts, the threat of third-party punishment is a clear constitutive part of a commitment and this had great effects for the extent of human cooperation. It is no surprise that changes in our cooperative landscape due to agriculture and organisational hierarchy had important consequences for human cooperation.

However, they were also important in the transition from reputationally-enforced to contractually-enforced commitments and, as such, had pivotal consequences for the scope of human cooperation in more ways than previously recognised.

The fact that there are so many different potential forms of institutionalised commitment makes it harder to determine and to date the conditions for their emergence in history, but not impossible. I have offered a discussion of the kinds of changes in our cooperative problems and organisation which would have facilitated the strengthening of our means of third-party punishment. Though the conditions will be different in different cultures at different times, this represents a first attempt at a narrative account of the emergence of this new enforcement mechanism. It also enables us to trace key steps in the emergence of institutionalised commitment, by looking at the emergence of agriculture, inequality, and social institutions in the empirical literature, as well as using agent-based models to determine their relationship.^21^ As we delve further into the deep past, such archaeological traces become harder to find. However, this does not mean the conditions for the emergence of institutionalised commitment are untestable, but that we may need to use proxies for certain data in order to verify and add credibility to the narrative presented.

One might object that a narrative of this sort is only a “just-so” story, positing the origins of a behaviour or trait without any testable implications (Gould & Lewontin, 1979). While this hypothesis involves some degree of uncertainty, there is a large middle-ground between fact and mere speculation. Indeed, others have argued for the benefits of narrative accounts and the conditions on what makes a good narrative account versus a bad one (Sterelny & Planer, 2021; Sterelny, 2021; Currie & Sterelny, 2017). I will not recapitulate this debate here. Rather, it is important to note that I have put forth a *hypothesis*, not fact, and that offering such hypotheses is a crucial part of developing our understanding of our social world. It is these hypotheses which generate testable predications for human behaviour. The empirical claim to be tested is whether institutionalised third-party punishment is reliant on such changes in our cooperative environment with delayed return on investment, sedentary lifestyles and organisational changes that promote hierarchy. The evidence that bears on this hypothesis is discussed above.

Today, with institutionalised forms of commitment readily available to many agents, we have copious means of making credible and low-cost commitments to cooperation with less risk of exploitation. Humans exhibit numerous examples of cooperative behaviour on a daily basis. While some animals have cooperative social lives too, humans are often spontaneously cooperative to those with whom they have never before interacted and, as the literature on ultimatum games shows, when there are no reputational benefits to be gained. Part of the explanation, I hold, lies in the proliferation of safe means of cooperation over our evolutionary history due to the development of increasingly effective commitments. The more cooperation is safeguarded by commitment, the less risky cooperation becomes.

## Data Availability

Not applicable.

## Footnotes

1 See West et al. (2007) for a taxonomy.

2 See Hamilton (1964).

3 Of course, an agent can also commit to choices which would not be better for her in the long run and can commit where she does not have an incentive to cheat in the short-term, but these will not be the cases of interest for explaining the evolution of cooperation.

4 An analogous argument can be made for the repeated Prisoner’s Dilemma where there is a cost to defection. See Skyrms (2003) for a discussion of how repeated interaction in the Prisoner’s Deilmma will serve to transform it into a Stag Hunt.

5 It is important to note that one could describe partner choice and partner control as two sides of the same coin. Indeed, Wubs et al. (2016) argue that the three main partner control mechanisms are: “(i) to switch from cooperation to defection when being defected (‘positive reciprocity’), (ii) to actively reduce the payoff of a defecting partner (‘punishment’), or (iii) to stop interacting and switch partner (‘partner switching’)” (Wubs et al., 2016: 1).

6 Of course this will not be true of all institutionalised commitments. For example, taking a landlord to court for not providing responsible care of one’s property is likely to be a costly and time-consuming process. In these cases, access to reputation may serve to bolster trust in new partners. Nonetheless, the complexity of this interaction is better safeguarded by means of institutionalised commitment than it is without.

7 For a discussion on this, see Boyd et al. (2003).

8 Hawks and Doves act differently with respect to contesting ownership rights to a territory. Doves posture and then each assumes the territory with equal probability. When a Dove meets a Hawk, the Hawk assumes the territory. When two Hawks meet, the value of the territory is less than the cost of the contest. Maynard Smith (1972) showed that there is an evolutionarily stable strategy when all agents behave like Hawks when they first find the territory and Doves otherwise. This is called the Bourgeois strategy. Gintis’ (2007) amendment to this model is to consider the resources devoted to the contest as endogenously determined, where the level of resources committed determines the probability of success. Interestingly, he notes this requires commitment, since without commitment, the agent might not continue to contest when the costs of doing so exceed her fitness benefit.

9 For more, see Fry (2000).

10 See also Bowles and Fochesato (2024) and Arnold et al. (2016), who argue that the purported relationship between agriculture and complexity is oversimplified.

11 See Feinmen et al. (2025) for a thorough discussion. See also Gurven et al. (2010) for a study of inequality in modern horticulturalists.

12 See Keeley (1988) for a defence of the population pressure position.

13 For a game-theoretical argument in favour of this, see Smith and Choi (2007). Also Hooper (2018).

14 See also Gurven et al. (2010) for a study of inequality in modern horticulturalists.

15 See, for example, Boyd and Richerson (1985).

16 See also Keen (2006) and Hayden (2018).

17 See Bowles and Fochesato (2024) for scepticism of the idea that religion played a central role in the emergence of inequality.

18 See Hooper (2018) for another computational model that shows hierarchy and inequality develop when coalitions form to defend resources. Inequality and institutionalisation here is taken to be a result of both ecological changes.

19 Note also that there are certain credibility-conferring mechanisms that are not based on punishment. Belonging to a religious community, even where it does not directly commit an agent to a particular course of action, can make one the *kind* of person who can swear credibly (Schelling, 2003). The all-encompassing nature of some religious practices mean that opportunities for observing someone’s faith are high, especially since many religious practices are demonstrated through public ritual – for example, going to church, having a bar mitzvah, praying in the mosque, or abstaining from worldly pleasures. Though these acts do not directly increase the probability of detecting defection for any particular oath, they do mean that a receiver’s credence in the reliability of an agent’s oath is greater in virtue of signalled religiosity. Of course, one’s action may not be as visible as it will be in a small-scale society, but this is not to say that institutionalised religion does not make one’s actions more observable than they would be otherwise.

20 See Brusse (2020) for a discussion of the difference between hard-to-fake, costly-to-fake and impossible-to-fake signals. For my purposes, it will not matter much whether the signal is impossible, difficult, or costly to fake. The important part is that there is some intrinsic feature of the activity which ensures only honest agents signal. This will be contrasted with cases where this is no such intrinsic feature, and honesty is maintained by externally-imposed costs.

21 See, for example, Anderson (1999) for a volume on the prehistory of agriculture and our methodological approaches to evidence-gathering and Beisaw and Gibb (2009) for an edited volume on the archeology of institutions. For modelling approaches, see Hooper et al. (2010) and Ozono et al. (2016) mentioned earlier, among others.
